# pH-Responsive Delivery of Nonsteroidal Anti-Inflammatory Drugs by ZIF‑8 Metal-Organic Frameworks for Cancer Treatment

**DOI:** 10.1021/acsomega.6c06939

**Published:** 2026-10-21

**Authors:** Nicola di Nicola, Mauro Di Vito Nolfi, Alice Vetrano, Jennifer Esposito, Patrizio Campitelli, Alessia Marino, Francesca Zazzeroni, Leonardo Guidoni, Marcello Crucianelli, Davide Vecchiotti, Andrea Lazzarini

**Affiliations:** † Department of Physical and Chemical Sciences (DSFC), 9303 University of L’Aquila, via Vetoio (“A.C. De Meis” building), Coppito, L’Aquila 67100, Italy; ‡ Consorzio INSTM UdR L’Aquila, via Vetoio (“A.C. De Meis” building), Coppito, L’Aquila 67100, Italy; § Department of Biotechnological and Applied Clinical Sciences (DISCAB), 9303 University of L’Aquila, via Vetoio (“A.C. De Meis” building), Coppito, L’Aquila 67100, Italy

## Abstract

Nonsteroidal anti-inflammatory drugs (NSAIDs) have attracted increasing attention as potential anticancer agents due to their ability to inhibit cyclooxygenase-mediated inflammatory pathways involved in tumor progression. However, their clinical use in oncology remains limited by poor tumor selectivity and systemic toxicity. In this work, we investigate zeolitic imidazolate framework-8 (ZIF-8) nanoparticles as pH-responsive carriers for the delivery of different NSAIDs with potential anticancer activity. A loading strategy based on the conversion of the drugs into their sodium salts, followed by ultrasound-assisted encapsulation, was developed to preserve the structural integrity of the MOF while achieving efficient drug incorporation. Structural and textural characterization by XRPD, SEM, ATR-MIR spectroscopy, and nitrogen physisorption confirmed the formation of ZIF-8 nanoparticles with an average size of about 150 nm and the successful encapsulation of the drugs within the porous framework. High-performance liquid chromatography revealed drug-loading efficiencies exceeding 80% for selected formulations. Release studies demonstrated a pronounced pH-dependent behavior, with significantly faster drug release under mildly acidic conditions that mimic the tumor microenvironment. The biological activity of selected formulations was evaluated in prostate-derived cell models (BPH, LNCaP, and DU145), revealing enhanced cytotoxicity toward cancer cells under acidic conditions compared to physiological pH. In particular, naproxen@ZIF-8 showed a marked reduction in DU145 cell viability at low concentrations and under mildly acidic conditions. These results highlight the potential of ZIF-8 as a versatile nanoplatform for the controlled and tumor-responsive delivery of anti-inflammatory drugs with an anticancer activity.

## Graphical abstract

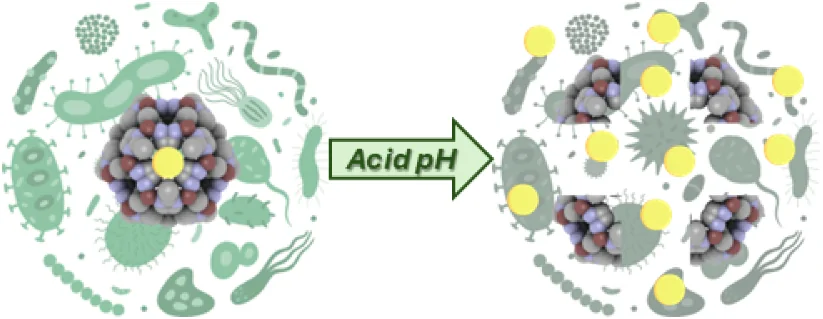

## Introduction

Cancer remains one of the leading causes of mortality worldwide, and despite significant advances in oncological research, treating this disease continues to pose considerable challenges.^1^ Traditional therapies, such as chemotherapy and radiotherapy, are often limited by severe side effects and poor specificity toward tumor tissues, reducing therapeutic efficacy and compromising patients’ quality of life.^2^ To address these issues, research has increasingly focused on the development of more targeted therapeutic approaches,^3^ including controlled drug delivery systems and pharmacological agents capable of simultaneously targeting multiple pathological mechanisms associated with tumor progression.^4^ In this context, Nonsteroidal Anti-Inflammatory Drugs (NSAIDs) have gained attention for their potential applications in oncology.^5^ Originally developed to treat inflammation and pain, NSAIDs have demonstrated antitumor properties in various preclinical and clinical studies.^6^ Their mechanism of action primarily involves the inhibition of cyclooxygenases (COX), key enzymes in the synthesis of prostaglandins, which play a central role in chronic inflammatory processes and tumorigenesis.^7^ Chronic inflammation is now recognized as a “hallmark” of cancer,^8^ as it promotes uncontrolled cell proliferation, angiogenesis, and tissue invasion.^9^ However, the long-term clinical use of NSAIDs is limited by systemic toxicity,^10^ including gastrointestinal damage, cardiovascular risks, and nephrotoxicity, highlighting the need for strategies to optimize their delivery and selectivity.^11^ The use of nanomaterials as drug delivery carriers offers a promising solution to these challenges.^12^ Nanocarriers are particularly advantageous due to their ability to improve drug biodistribution, enhance accumulation in tumor tissues via the enhanced permeability and retention (EPR) effect,^13^ and minimize systemic side effects.^14^ Among the various types of nanomaterials, Metal-Organic Frameworks (MOFs) have emerged as highly versatile platforms due to their unique structural and functional properties.^15^ MOFs are hybrid crystalline materials composed of metal nodes coordinated with organic linkers, forming a three-dimensional porous network.^16^ This peculiarity makes them ideal for encapsulating bioactive molecules and releasing them in a controlled manner in response to specific stimuli, such as pH, temperature, or enzymatic activity.^17^ Among the most studied MOFs, the Zeolitic Imidazolate Framework-8 (ZIF-8) stands out for its exceptional chemical and mechanical stability, biocompatibility, and pH-responsive drug release capability.^18^ ZIF-8 is composed of zinc metal nodes coordinated with imidazolate linkers, forming a highly stable crystalline lattice. This structure allows for the effective encapsulation of both hydrophobic and hydrophilic molecules, protecting them from premature degradation and ensuring controlled release in acidic environments, such as those typically found in solid tumors.^19^ Additionally, ZIF-8 can be easily functionalized to enhance tumor cell specificity or integrate additional therapeutic and diagnostic features.^20^ While the potential of ZIF-8 as a carrier for antitumor drugs has been explored in several studies,^21^ the integration of NSAIDs into these nanostructured systems remains a relatively less investigated area of research. NSAIDs, due to their ability to modulate inflammation and interfere with tumor growth mechanisms,^22^ could significantly benefit from encapsulation in ZIF-8. This approach would enable targeted and controlled drug release while reducing systemic toxicity.^23^ In this study, we aim to investigate the potential of ZIF-8 as a carrier for the delivery and controlled release of NSAIDs with antitumor activity. The main objectives of our study are hereby summarized: evaluate the encapsulation efficiency and stability of the ZIF-8-NSAID system, analyze the drug release kinetics under simulated physiological and pathological conditions, and assess the biological activity of the system in vitro on cancer cell lines. The findings of this research could provide a foundation for developing new therapeutic strategies that combine the versatility of MOFs with the antitumor properties of NSAIDs, ultimately improving the efficacy and safety of cancer treatments. By integrating NSAIDs into ZIF-8, this approach has the potential to offer a significant advancement in the design of targeted therapies, paving the way for innovative solutions in cancer treatment.

## Experimental Section

### Materials

Zinc nitrate hexahydrate (98%), 2-methylimidazole (MeIm, 99%), acetylsalicylic acid (98%), acetaminophen (99%) (paracetamol), 4-isobutyl-alpha-methylphenylacetic acid (>98%) (ibuprofen), 2′-fluoro-alpha-methyl-4-biphenylacetic acid (>98%) (flurbiprofen), 2-(3-benzoylphenyl)­propionic acid (>98%) (ketoprofen), 2-(6-methoxy-2-naphthyl)­propanoic acid (97%) (naproxen), methanol (99.8%), acetonitrile (>99.9%), sodium hydroxide, acetic acid (99.7%), phosphate-buffered saline tablets (PBS, 0.0027 M KCl; 0.137 M NaCl; pH = 7.4) were purchased from Sigma-Aldrich. Doubly deionized water was obtained from a Milli-Q filtration/purification system (Millipore, Bedford, MA, USA).

### Characterization Techniques

#### X-ray Powder Diffraction (XRPD)

Diffraction patterns were collected using a PANalytical EMPYREAN diffractometer operating in the Bragg–Brentano geometry with Cu Kα radiation (λ = 1.5418 Å) over a 2θ range of 3° to 40° (step size: 0.013°; time per step: 30 s; Soller slit: 0.04 rad; divergence slit: 1/8; antiscatter slit: 1/4; 45 mA × 40 kV). Measurements at room temperature were conducted using a spinning silicon zero-background sample holder.

#### Attenuated Total Reflectance Mid-Infrared Spectroscopy (ATR-MIR)

ATR-MIR measurements were performed with a PerkinElmer Spectrum Two instrument equipped with an UATR Two accessory and a DTGS detector. Each spectrum was obtained by averaging 8 scans at a 4 cm^–1^ resolution in the range 4000–400 cm^–1^.

#### Thermogravimetric Analysis (TGA)

TGA was used to determine the material’s stoichiometry and was performed with a Hitachi STA200RV instrument; the measurements were performed in synthetic air flow (50 mL/min) with a temperature ramp of 10 °C/min from r.t. to 750 °C.

#### Surface Area Analysis

Surface area measurements were performed with the use of an Anton Paar NOVA 800 instrument. The sample was inserted inside a calibrated-volume sample holder and treated at 150 °C in dynamic vacuum for 18 h directly on the instrument. Surface area was measured with N_2_ adsorption–desorption isotherms at 77 K by using the BET method.

#### Scanning Electron Microscopy (SEM)

The morphology of the samples was analyzed using scanning electron microscopy (SEM) with an FESEM ZEISS Gemini 500 instrument. Images were captured at 10k and 50k magnifications using backscattered electrons, operating at an accelerating voltage (HT) of 5 kV. The samples were mounted on SEM stubs covered with carbon tape and subsequently coated with a thin chromium layer to increase the conductivity and prevent electrical charging during imaging.

#### UV–vis Spectroscopy

UV–vis measurements were performed to evaluate the concentration of individual species in order to determine the release curve by determining the individual concentrations in solution as a function of time. The analysis was performed with an ONDA UV-30 SCAN spectrophotometer in the range of 200–350 nm. The concentrations of the different NSAID salts were quantified on the basis of the intensities of the absorption bands.

#### High-Performance Liquid Chromatography (HPLC)

Chromatographic analyses of pharmaceutically active compounds were carried out using an Agilent 1100 Series high-performance liquid chromatography (HPLC) system equipped with a diode array detector (DAD). Separation was achieved on a Phenomenex Luna C18 column (250 mm × 4.6 mm i.d., 5 μm particle size), operating under isocratic elution conditions. The mobile phase consisted of water and acetonitrile in an 80:20 (v/v) ratio, delivered at a constant flow rate of 1.0 mL/min. An injection volume of 2 μL was used for all analyses.

The analyzed solutions were obtained by mixing the acetonitrile solution from the sonicator, after removing the ZIF-8 by centrifugation, and the MeOH washing solution. All solutions were then made up to volume with acetonitrile in a 200 mL volumetric flask. Calibration curves of the individual NSAIDs were constructed using five solutions of each known concentration, prepared to cover a linear range of concentrations representative of the test sample. The peak area values obtained for each standard were plotted against the respective concentrations, and the calibration line was determined by linear regression of the data. The concentration of the active principle in the sample was then determined by interpolating the peak area value on the calibration line (Table S1).

The detection wavelength was selected individually for each compound based on its specific UV–vis absorbance profile, allowing optimal sensitivity and selectivity. This setup ensured reproducible retention times and reliable quantification of target analytes.

### ZIF-8 Synthesis

The synthesis of ZIF-8 has been extensively studied, with various methods reported to achieve slight structural modifications. In this study, we adapted the hydrothermal synthesis procedure developed by Koji Kida and collaborators,^24^ particularly suitable for biomedical applications due to the use of water as a solvent, while scaling up the synthesis to produce gram-scale quantities of the material. Specifically, 1.488 g of zinc nitrate hexahydrate was dissolved in 40 mL of deionized water and subsequently combined with a solution containing 24.6 g of 2-methylimidazole (MeIm) in 160 mL of Milli-Q water. The reaction mixture, which rapidly became turbid, was stirred at room temperature for 2 h. Following the synthesis, the suspension was centrifuged at 5000 rpm for 30 min and washed three times with methanol. The final product was dried overnight under reduced pressure at 40 °C.

### NSAID Salification

All selected NSAIDs, except for paracetamol, were converted into their corresponding sodium salt forms prior to the loading experiments. This approach was adopted to ensure a consistent chemical form of the investigated compounds and to avoid the partial degradation of ZIF-8 observed when the NSAIDs were directly loaded in their free-acid form. The sodium salts were prepared by reacting the corresponding NSAIDs with stoichiometric amounts of NaOH in water (0.05 M with respect to the active compound) for 3 h at 70 °C. Stoichiometric amounts of NaOH were employed to minimize the presence of unreacted base. Upon completion of the reaction, the solvent was removed by rotary evaporation and the resulting sodium salts were further dried overnight under vacuum at 70 °C. Under these reaction conditions, the neutralization of the carboxylic acid groups produces only water as the reaction byproduct and the use of stoichiometric base avoids the presence of unreacted drug. The successful conversion of the carboxylic acid groups into the corresponding carboxylate species was confirmed by ATR-MIR spectroscopy, which showed the disappearance of the characteristic bands associated with the COOH groups and the appearance of the characteristic carboxylate bands (see the Results and Discussion section and the Supporting Information).

### NSAID Loading

The loading process by sonication was performed using a pulsed probe sonicator, with a 1 s pulse and 3 s of rest (Sonics Materials VCX-500-220). A mixture of 0.88 g of previously synthesized ZIF-8 and 0.133 g of the active compound was dispersed in acetonitrile (at a concentration of 0.005 M relative to the NSAID). The suspension was sonicated for 30 min while being thermostated in an ice bath. The product was recovered by centrifugation at 5000 rpm for 30 min, washed once with methanol, and dried overnight at 40 °C under vacuum.

To calculate the percentage of drug-loading efficiency (%DLE; eq 1), we used the following equation:

1 $$\%\mathrm{DLE}=\frac{\mathrm{starting mass of the drug}-\mathrm{mass of the drug in the washing solutions}}{\mathrm{starting mass of the drug}}\times 100$$

To calculate the percentage of drug loading inside ZIF-8 (%DL; eq 2), we used the following equation:

2 $$\mathrm{\%DL}=\frac{\mathrm{starting drug mass}-\mathrm{drug mass in washing solutions}}{\left(\mathrm{starting drug mass}-\mathrm{drug mass in washing solutions}\right)+\mathrm{ZIF‐8 mass}}\times 100$$

The obtained materials were thoroughly characterized, and the results are reported in Figures S2–S6.

### Release Test

NSAID release tests were conducted by dispersing 100 mg of the previously synthesized NSAID@ZIF-8 system in a solution of 18 mL of acetate buffer (pH = 5.5) and 2 mL MeOH due to the material’s poor suspensibility in water. The same setup was used with PBS (pH = 7.4). The suspension was kept under gentle agitation at 37 °C, simulating average body temperature, for several days.

In order to evaluate the amount of the active molecule released, we employed the following equation, describing the percentage of Drug Release Efficiency (%DRE; eq 3) as a function of time:

3 $$\mathrm{\%DRE}=\frac{\mathrm{moles of drug released}}{\mathrm{moles of drug loaded}}\times 100$$

### Cell Culture and Viability Test

NSAIDs and different ZIF-8 formulations were tested on benign prostatic hyperplasia (BPH) cells and prostate cancer cell lines LNCaP and DU145. The BPH cell line was cultured in RPMI medium supplemented with 20% Fetal Bovine Serum (FBS) (Immunological Sciences), 1% penicillin/streptomycin (100 U/mL; 100 μg/mL) (Euroclone), and 1% l-glutamine (2 mM) (Euroclone), LNCaP in RPMI medium supplemented with 10% FBS, 1% penicillin/streptomycin, 1% l-glutamine, 1% sodium pyruvate (Lonza), and 1% HEPES (Euroclone), and DU145 in RPMI medium supplemented with 10% FBS, 1% penicillin/streptomycin, and 1% l-glutamine. All cell lines were cultured at 37 °C with 5% CO_2_ and viability was assessed with PrestoBlue Cell Viability Reagent (Invitrogen) in accordance with the manufacturer’s instructions. Viability values were read with a μQuant (BioTek Instruments).

### Cell Treatments

Prostate cell lines were cultured in 96-well plates and treated with different concentrations of the different compounds. Free drugs and ZIF-8 formulations were dissolved in methanol at concentrations of 150 mM and 2.5 mg/mL, respectively, and tumor microenvironmental acidity was mimicked adjusting pH of culture media at 6.8 with 1 M sterile-filtered HCl.

## Results and Discussion

### Synthesis, Loading, and Characterization

ZIF-8 is among the most extensively studied metal-organic frameworks (MOFs) in the biomedical field, with numerous synthetic approaches available for its preparation. In this study, ZIF-8 was synthesized, loaded, and characterized by following an optimized protocol previously developed by our research group.^25^ This method produces ZIF-8 crystals with an average size of approximately 150 nm and uniform morphology, as illustrated in Figure 1.

**Figure 1:**
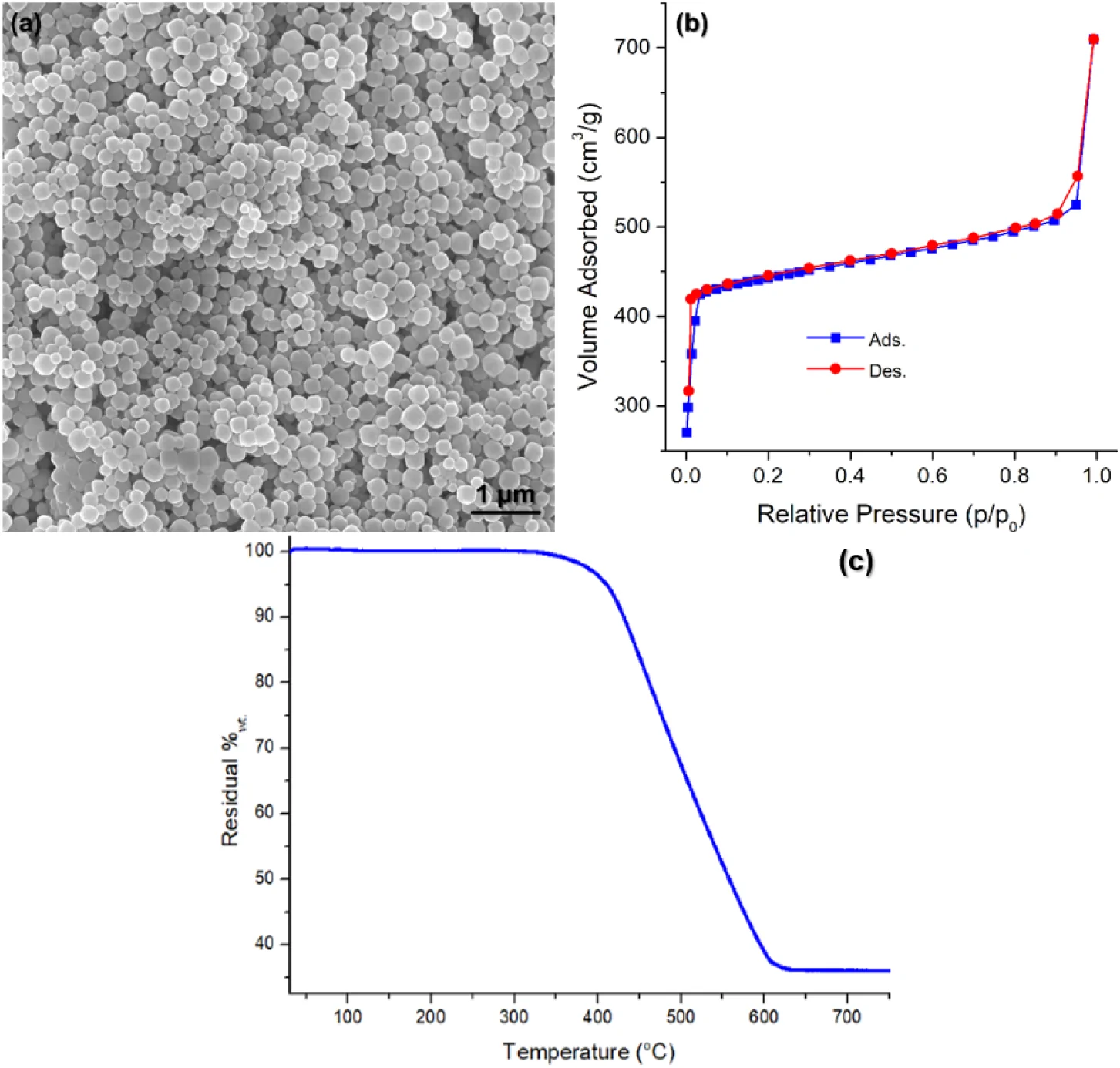
(a) ZIF-8 SEM image collected at 10 kx magnification. (b) N_2_ adsorption (blue)–desorption (red) isotherms at 77 K of ZIF-8. (c) TGA curve of ZIF-8.

The surface area, determined by nitrogen adsorption at 77 K, was found to be 1837 m^2^/g, confirming that the morphological properties of the MOF are consistent with those reported in the literature as well as the residual mass from TGA, confirming the preparation of ZIF-8 according to the correct stoichiometry.^26^ The next step was to optimize the loading of NSAIDs into the cavities of ZIF-8. Among the different possibilities, we selected acetylsalicylic acid, paracetamol, ibuprofen, flurbiprofen, ketoprofen, and naproxen, representing different sizes and functional groups that may be a challenge for drug uptake inside the MOF matrix. Ibuprofen was chosen as the reference compound to optimize loading conditions, as its size represents an average among NSAIDs intended for encapsulation within the MOF and reported throughout the paper; the same procedure was followed for the other NSAIDs, and the relative data are reported in the Supporting Information. Initially, an attempt was made to load the NSAIDs in their acid form in methanol via a stirring method. However, the presence of the carboxylic acid functional group, characteristic of all these compounds except for paracetamol, hindered the loading process by causing MOF decomposition.^27^ Instead, it led to the partial decomposition of the MOF, which is highly sensitive to pH, a property that is intended to be exploited in this application. The result was a significant decrease in yield, with only about 10% of the initial material recovered; for this reason, we decided to stop loading the active pharmaceutical ingredients in their free acid form and proceed instead with the respective sodium salts. The salification of these compounds is relatively simple because of the presence of carboxylic acid as the only reactive site. The reactions were conducted in the presence of stoichiometric amounts of NaOH.^28^

Once isolated as dry powders, the products were characterized by ATR-MIR spectroscopy. Although not a quantitative analysis, ATR-MIR is useful in identifying the nature of the products because of the characteristic peaks of the carbonyl and carboxylic groups. As shown in Figure 2 for the ibuprofen sample, comparison of the spectra of the active ingredient and its corresponding salt clearly shows the disappearance of the characteristic peaks at 1721 and 948 cm^–1^, corresponding to C=O stretching and −OH bending respectively, followed by the appearance of the double peak at 1580 and 1400 cm^–1^ typical of the symmetric and antisymmetric stretching of the newly formed carboxylate species.^29^ This confirms the complete consumption of the starting material, which has been quantitatively converted into salt.

**Figure 2:**
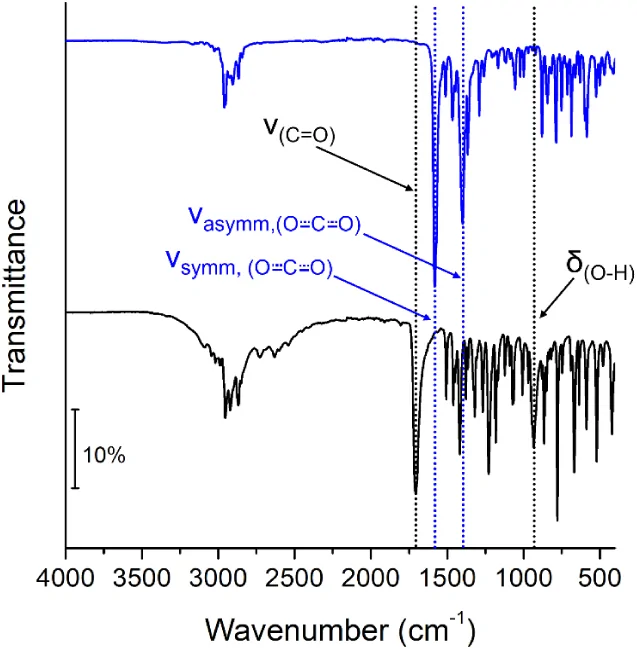
ATR-MIR spectra of ibuprofen (black line) and ibuprofen sodium salt (blue line).

Once all the drugs were converted into their salt forms, we returned to the primary objective of this study, i.e., the optimization of NSAID loading into ZIF-8. However, instead of using the conventional stirring method, we employed a probe sonicator for the loading process. The underlying principle of this approach is to utilize ultrasonic waves to solubilize the obtained salts in a solvent that is inherently unfavorable for these compounds, thereby making ZIF-8 a more suitable environment for their incorporation. To optimize this process, solubility screening was conducted in various solvents, including water, methanol, isopropanol, and acetonitrile. Among these, acetonitrile proved to be the most effective, as the selected NSAID salts exhibited poor solubility in this solvent, favoring their entrapment within the ZIF-8 framework (Figure S1a).

This approach was applied to all NSAIDs under study, and the resulting materials, i.e., ZIF-8-containing drugs, were thoroughly characterized. XRPD analysis confirmed the structural integrity of the SOD crystal structure (Figure 3d),^18^ while SEM (Figure 3b) and ATR-MIR spectroscopy (Figure 3a) verified the encapsulation of the active pharmaceutical ingredients within ZIF-8 and the superficial structural integrity.^25^ In addition, physisorption of nitrogen at 77 K revealed a reduction in surface area (1447 m^2^/g), indicative of the effective incorporation of molecules into the host (Figure 3c and Table S2 for comparison with other materials). This is also confirmed by the maintained shape of the isotherms, indicative of the porous structure of the material that stayed untouched. The surface area values calculated using the BET equation (see the Supporting Information) confirm the effective presence of the drugs within the pores of ZIF-8. The discrepancy between the surface area value of the pure MOF and the different materials obtained results in pore occlusion caused by the presence of the drugs.

**Figure 3:**
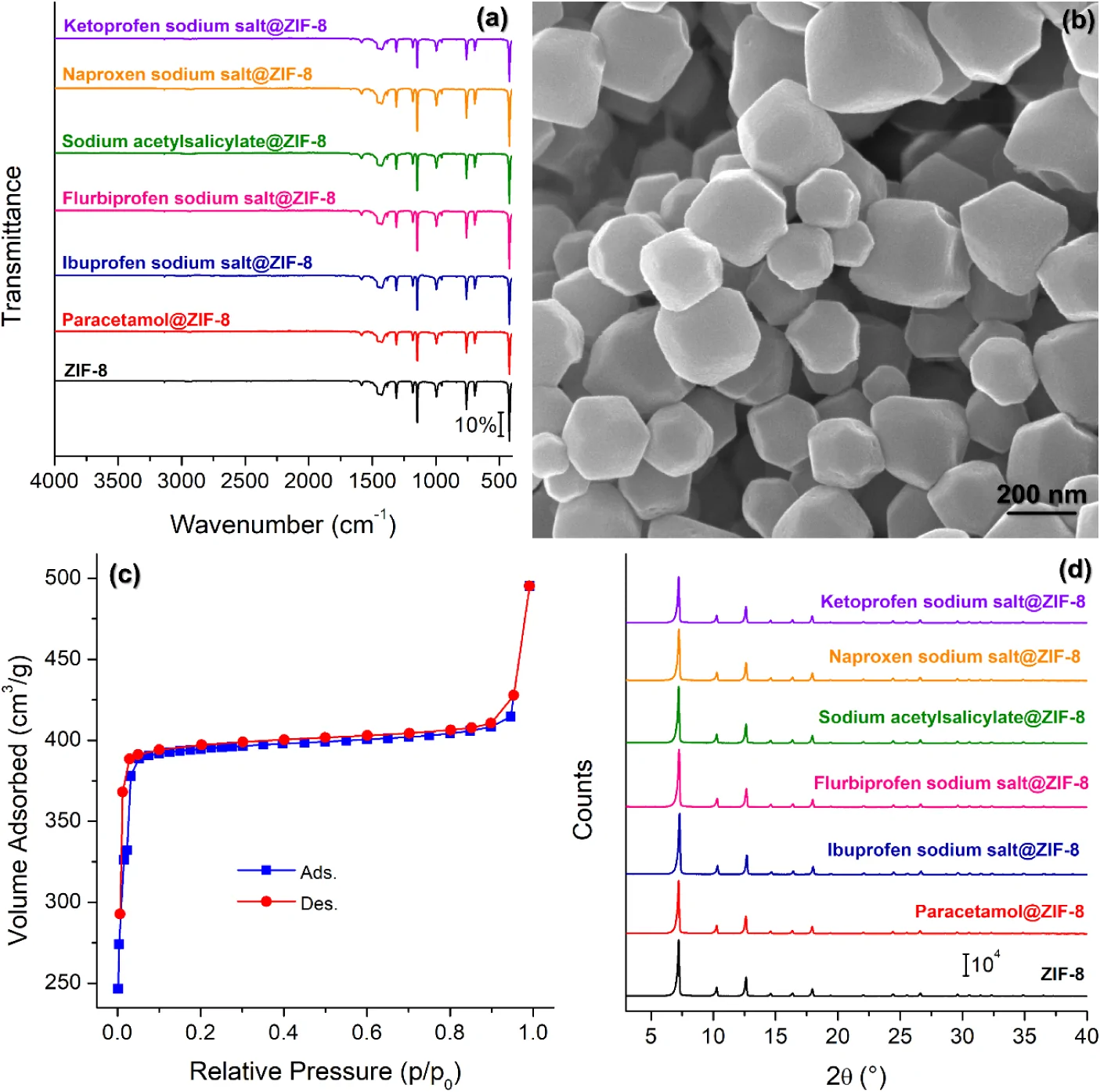
(a) Comparison of the experimental ATR-MIR of pure ZIF-8 and ZIF-8 loaded with different NSAIDs. (b) SEM image collected at 50 kX of the ibuprofen@ZIF-8 system. (c) N_2_ adsorption–desorption isotherms at 77 K of ibuprofen@ZIF-8. (d) Comparison of the experimental XRPD pattern of pure ZIF-8 and different ZIF-8 loaded with different NSAIDs.

In order to quantify the individual active principles loaded within the porous structure, measurements were carried out by HPLC. The drug loading (DL) and drug loading efficiency (DLE) percentage values, shown in Table 1, demonstrate how efficient the use of the pulsed sonicator to load the NSAIDs used is, leading in some cases to a loading efficiency of over 80%. The materials obtained showed a high percentage of drug, demonstrating the ability of ZIF-8 to trap molecules of different dimensions.

**Table 1:** Data from HPLC Analysis and DLE% and DL% Values

	DLE%^a^	DL%^b^	area	slope	R^2^
**Ibuprofen sodium salt**	73.2	52.5	854.96	4801.9	0.999
**Sodium acetylsalicylate**	93.4	58.5	89.94	1994.2	0.999
**Flurbiprofen sodium salt**	66.9	50.3	1398.25	6359.2	0.999
**Ketoprofen sodium salt**	64.7	49.4	1431.89	6090.7	0.999
**Naproxen sodium salt**	58.5	46.9	1224.81	4438.7	0.999
**Paracetamol**	81.6	55.2	500.79	8200.9	0.999

a Calculated according to eq 1.

b Calculated according to eq 2.

The use of ultrasound plays a fundamental role in this process, forcing the drugs under investigation to enter the cavities of the crystal, a more favorable environment than that of acetonitrile in which they are poorly soluble or insoluble.

The different loading efficiencies observed for the investigated NSAIDs can be qualitatively related to their distinct molecular structures and physicochemical properties. The compounds differ in molecular size, functional groups, polarity and, consequently, in their solubility in the acetonitrile loading medium. These factors can affect the ability of the sodium-salt forms to be transferred from the solvent phase into the porous environment of ZIF-8 during ultrasound treatment. In particular, the DLE values range from 58.5% for naproxen sodium salt to 93.4% for sodium acetylsalicylate, while the corresponding DL values are confined to a narrower range (46.9–58.5%). This indicates that the molecular structure influences the efficiency of the loading process, but does not result in a pronounced difference in the final amount of drug incorporated into the ZIF-8 framework. Since the solubility of the individual sodium salts in acetonitrile was not quantitatively determined, a direct structure–loading correlation cannot be established; nevertheless, the observed differences are consistent with the combined influence of molecular size and physicochemical properties on the loading process.

### Release Tests

The final step of this work is to observe the release behavior of the diverse ZIF-8 systems with each NSAID encapsulated within it, under different pH conditions (according to eq 3). The curves reported in Figure 4 derived from various concentration values obtained through UV–vis spectroscopy, clearly illustrating the different release behaviors of ibuprofen sodium salt (for other release tests, consult the Supporting Information) depending on the pH value. Indeed, in an acidic environment, 94% of the drug is released within 24 h, while at pH 7.4, only 62% of the encapsulated active principle is released in the same period. Under acidic conditions, the release rapidly reaches a plateau after 36 h, achieving a total drug release of 95.7%. In contrast, to reach the same level of release under a neutral pH, a period of 7 days is required.

**Figure 4:**
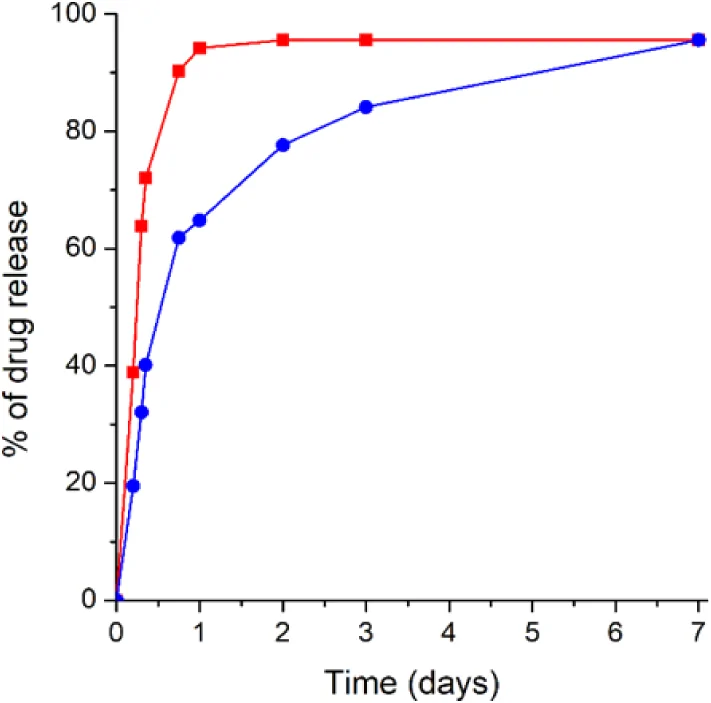
Time-dependent release profile of ibuprofen at pH 5.5 (red line) and pH 7.4 (blue line).

The pH-dependent release behavior can be related to the chemical stability of the ZIF-8 framework under the investigated conditions. In our previous study on sulfathiazole@ZIF-8, the stability of pristine ZIF-8 was directly investigated by time-dependent XRPD and SEM analyses at pH 5 and 7.4. The framework was stable under physiological conditions, whereas at pH 5, its degradation was already detectable after 24 h and was complete within 48 h.^25,30^ The faster drug release observed under mildly acidic conditions in the present work is therefore consistent with the acid-induced degradation of the ZIF-8 framework. Conversely, the slower release observed at pH 7.4 is consistent with the higher stability of ZIF-8 under physiological conditions. Notably, similar pH-dependent release profiles were observed for all of the investigated NSAIDs (Figures S2–S6), supporting a common carrier-controlled release mechanism rather than a behavior specific to the chemical nature of the individual drugs.

These findings indicate a pH-dependent drug-release mechanism that is consistent with the pH-responsive stability of the ZIF-8 framework. For a generic tumor model, a faster release of ibuprofen is anticipated in the acidic regions of tumors. This pH-responsive behavior could be particularly beneficial during early-stage treatment by enhancing the release of the anti-inflammatory agent in response to localized acidification, potentially modulating COX-2 activity in the tumor tissues.

### Biological Tests

Inflammation plays a key role in cancer development and progression. NF-κB signaling is a master regulator of inflammation,^31^ drives COX-2 transcription,^32^ and its upregulation is associated with prostate cancer progression and poor prognosis.^33^ Moreover, NF-κB activation correlates with androgen insensitivity, thus affecting prostate cancer aggressiveness that increases from androgen sensitive (AS) to androgen insensitive (AI) tumors.^34^ Although inhibition of inflammatory pathways and COX enzymes is an excellent strategy to affect cancer cell survival,^35^ it is limited by poor selectivity and systemic side effects, thus imposing a more specific approach such as a proper delivery system with a localized release mechanism. Considering the role of inflammation in prostate cancer and relative expression levels of COX-1 and COX-2 in different prostatic pathologies, we tested our ZIF-8 formulations on BPH, LNCaP, and DU145 cell lines. The nontumor BPH cell line represents a model of benign prostatic hyperplasia, while LNCaP (AS) and DU145 (AI) are considered models of prostate carcinoma with different grades of aggressiveness. More specifically, the DU145 cell line represents the more aggressive cancer model and shows a more marked activation of NF-κB signaling compared to LNCaP.^33^ Since NSAIDs show different selectivity in inhibiting COX-1, COX-2, and NF-κB pathway, we chose three ZIF-8 formulations for cell treatment, specifically Sodium Acetylsalicylate@ZIF-8, Ibuprofen@ZIF-8, and Naproxen@ZIF-8; while Ibuprofen and Naproxen are more selective in inhibiting COX-2, Sodium Acetylsalicylate is more selective for COX-1. To mimic the acidity of the tumor microenvironment, prostate cell lines were treated both at physiological pH (7.4) and at mildly acidic pH (6.8), and viability was assessed after 96 h. Although the microenvironment of BPH is not acidic, we treated BPH cells also in acidic pH for clarity. As shown in Figure 5, all formulations have a greater efficacy at acidic pH, confirming that the release of drugs is pH-dependent. As expected, the viability of BPH cells is not affected by Ibuprofen@ZIF-8 and Naproxen@ZIF-8; notably, most probably due to the expression of COX-1 in BPH,^36^ viability decreases only at relatively high doses of Sodium Acetylsalicylate@ZIF-8, suggesting that Sodium Acetylsalicylate could be a good candidate in the treatment of BPH disease. On the other hand, the viability of LNCaP is dependent on the concentration of ZIF-8 formulations; although an effect was also observed at physiological pH, the selected formulations showed better performance at acidic pH, Ibuprofen@ZIF-8 particularly. Best results were obtained in the most aggressive prostate cancer cell line, DU145; although Sodium Acetylsalicylate@ZIF-8 significantly decreases cell viability, Naproxen@ZIF-8, the most selective toward COX-2 isoform, completely suppresses cell viability at pH 6.8 and at a low concentration (20 μg/mL). Importantly, the viability of prostate cancer cell lines treated with free drugs decreased only at very high concentrations (Figure S7), confirming the efficacy of MOFs as drug delivery systems. Overall, no significant effect was observed in cells treated with vehicle (Methanol) and ZIF-8 alone. In summary, these data show that different NSAIDs have a specific efficacy on different prostatic pathologies and that MOF-encapsulating formulations can be selective for cancer cells that are characterized by an acidic microenvironment compared to normal tissues. Specifically, the different and selective effects of various formulations among different cell lines are closely related to relative COX enzyme expression and NF-κB activation.

**Figure 5:**
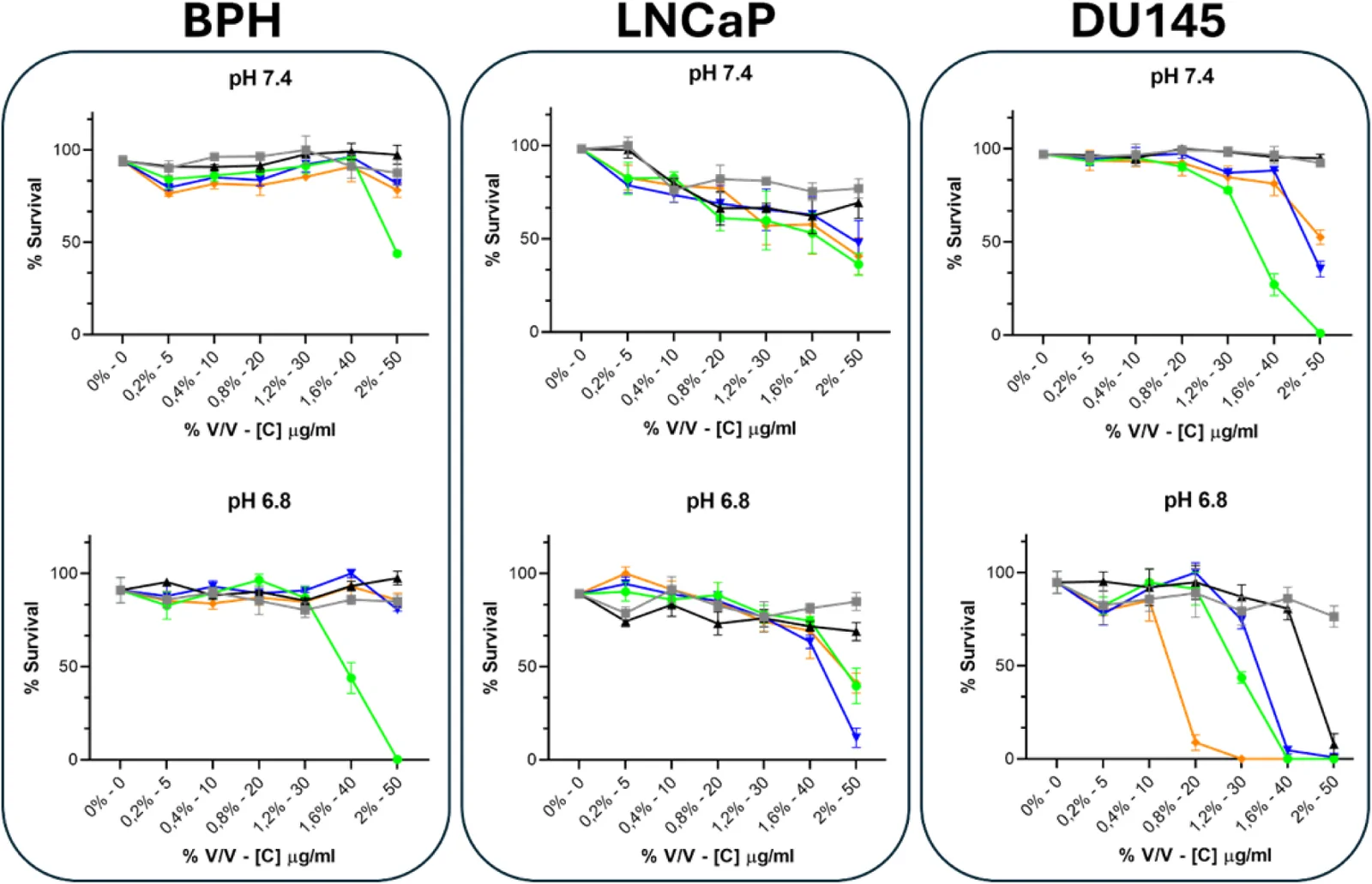
PrestoBlue viability assay on prostate cell lines (BPH as a model of benign prostatic hyperplasia; LNCaP and DU145 as less and more aggressive prostate carcinoma, respectively). ZIF-8 formulations (as prepared: black; sodium acetylsalicylate-loaded: green; ibuprofen-loaded: blue; naproxen-loaded: orange) were tested at a concentration range varying from 0 to 50 μg/mL, and controls were treated with the same %V/V of methanol (vehicle: gray).

## Conclusions

This study demonstrates once again the effectiveness of ZIF-8 as a nanoplatform for the encapsulation and delivery of drugs, and specifically of nonsteroidal anti-inflammatory drugs (NSAIDs) in this case for prostate cancer treatment. The ultrasound-assisted loading strategy based on sodium salt formation proved to be a robust and versatile approach, enabling high drug incorporation while preserving the structural integrity of the framework. The obtained systems exhibit clear pH-responsive release behavior, promoting preferential drug delivery under mildly acidic conditions that mimic the tumor microenvironment.

This pH-dependent release translates into enhanced biological activity in prostate cancer models, highlighting the potential of ZIF-8 to improve the therapeutic profile of NSAIDs. Specifically, the ability of ZIF-8 to respond to the tumor microenvironment suggests that it could be employed to enhance the therapeutic efficacy of NSAIDs by modulating the COX-1 and COX-2 activities in tumor tissues. More specifically, although COX-1 is constitutively expressed in many tissues, COX-2 is an inducible isoform and is highly expressed in inflammatory diseases such as cancer via NF-κB activation.^37^ Notably, Naproxen@ZIF-8 exhibited a remarkable anticancer effect against the aggressive DU145 cell line, leading to a complete suppression of cell viability at low concentrations under mildly acidic conditions. This result underscores the potential of combining COX-2 inhibition with pH-triggered delivery for the selective targeting of advanced prostate cancer. In addition, acidosis in the tumor microenvironment triggers the activation of inflammatory pathways and upregulates COX-2 expression,^38^ thus enhancing the specificity and the pharmacological effect of delivered NSAIDs.

Furthermore, as observed in sodium acetylsalicylate@ZIF-8-treated BPH cells, specific NSAIDs can also be employed for other prostatic pathologies. This aspect is most probably due to the variable selectivity of NSAIDs toward COX-1 and COX-2 and to differential expression of COX isoforms in the various pathologies and can be very useful in developing a highly specific targeted therapy.

Overall, these findings highlight the potential of ZIF-8-based systems to enhance the efficacy of repurposed anti-inflammatory drugs and support further investigation of these nanoplatforms for targeted cancer therapies. Future studies will focus on in vivo evaluation and further elucidation of the release mechanisms and biocompatibility of the system.

## Supplementary Material



## Data Availability

The data supporting this article have been included as part of the Supporting Information.

## Notes

a) UV–vis spectra of ibuprofen washing solutions in isopropanol, methanol, and acetonitrile and b) UV–vis spectra of the NSAIDs used, dissolved in acetonitrile (Figure S1); a) ATR-MIR spectra, b) N2 adsorption–desorption isotherms at 77 K, c) SEM image collected at 50 kx, and d) time-dependent release rate trends at pH 5.5 (red line) and pH 7.4 (blue line) for the different NSAIDs employed in this study (Figures S2–S6); PrestoBlue viability assay on LNCaP (top) and DU145 (bottom) prostate cancer cell lines treated with free drugs (Figure S7); spectroscopic data from HPLC analysis (Table S1); BET surface area for the as-prepared ZIF-8 and the drug-loaded ones (Table S2) (PDF)
