# Supplementary material for: pH-Responsive Delivery of Nonsteroidal Anti-Inflammatory Drugs by ZIF‑8 Metal-Organic Frameworks for Cancer Treatment
Source: ACS Omega. 2026 Oct 21;11(43):66933–41. doi: 10.1021/acsomega.6c06939 (PMC13736975; doi:10.1021/acsomega.6c06939)
Supplement: Supplementary file 1 [file ao6c06939_si_001.pdf]

Supporting Information

pH-responsive Delivery of Non-Steroidal Anti-Inflammatory Drugs by ZIF-8 Metal-Organic Frameworks for Cancer Treatment

Nicola di Nicola[ab], Mauro Di Vito Nolfi[c], Alice Vetrano[a], Jennifer Esposito[a], Patrizio Campitelli[a], Alessia Marino[a], Francesca Zazzeroni[c], Leonardo Guidoni[a], Marcello Crucianelli[ab], Davide Vecchiotti[c], Andrea Lazzarini[ab*]

[a] *Department of Physical and Chemical Sciences (DSFC), University of L'Aquila, Via Vetoio ("A.C. De Meis" building), Coppito, L'Aquila, 67100, Italy.*
[b] *Consorzio INSTM UdR L'Aquila, Via Vetoio ("A.C. De Meis" building), Coppito, L'Aquila, 67100, Italy.*
[c] *Department of Biotechnological and Applied Clinical Sciences (DISCAB), University of L'Aquila Via Vetoio ("A.C. De Meis" building), Coppito, L'Aquila, 67100, Italy.*
* Corresponding author - email: andrea.lazzarini@univaq.it

The choice of solvent to be used in the NSAID loading study was evaluated by UV-Vis analysis of the washing solutions. This short solvent screening showed that the use of acetonitrile as a solvent for loading tests leads to a lower concentration of the active principle in the washing solutions (Figure S1a), and therefore a higher amount within the ZIF-8. UV-Vis has also been used to identify the λ_{max} (nm), necessary for the HPLC analysis (Figure S1b).

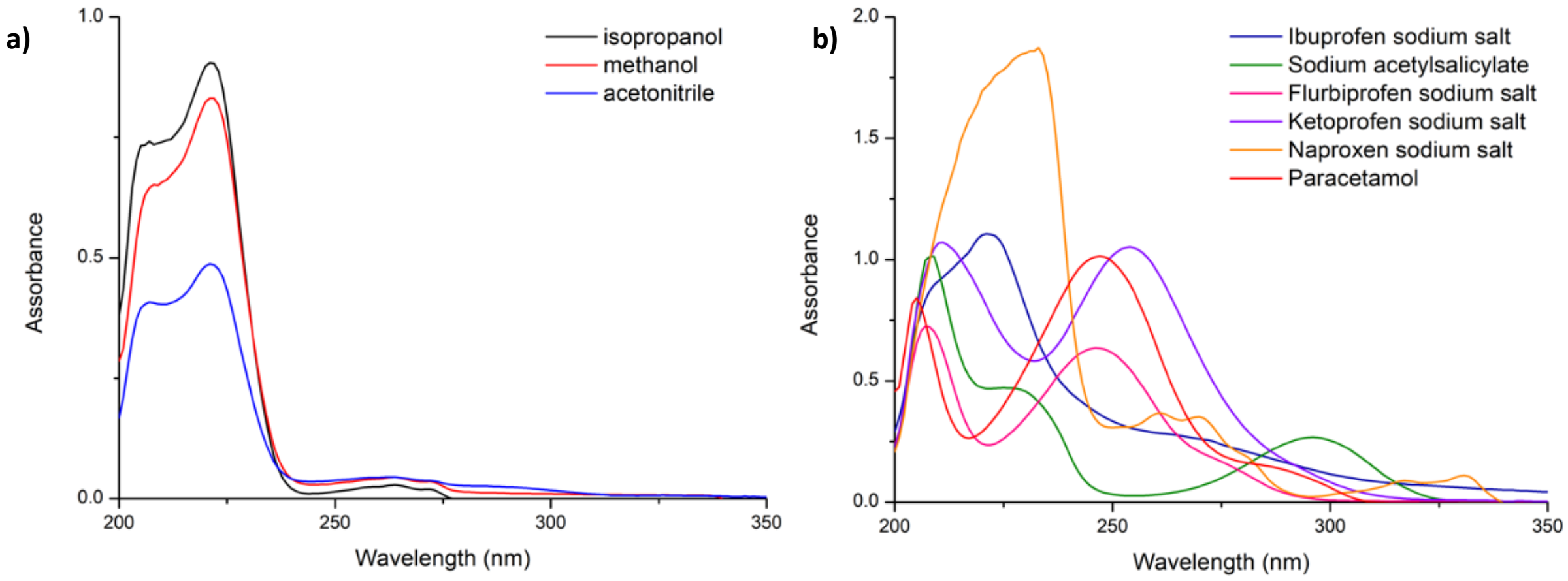

Fig. S1. a) UV-Vis spectra of ibuprofen washing solutions in isopropanol, methanol and acetonitrile. b) UV-Vis spectra of NSAIDs used, dissolved in acetonitrile.

In addition, UV-Vis spectrophotometry was used to evaluate trends in drug release tests. The calibration curves for individual NSAIDs were constructed using five solutions of known concentration, prepared to cover a linear range representative of the sample under consideration. The measured absorbance values for each solution were plotted against the corresponding concentration and the calibration curve was obtained by linear regression analysis (Table S1). The concentration of the pharmaceutical active substance was then determined by interpolating the measured absorbance value in the resulting calibration curve.

Table S1. Spectroscopic data from HPLC analysis.

	**λ_{max} (nm)**	**ε_λ (m^2/mol)**	**R^2**
**Ibuprofen sodium salt**	221	1.10×10^3	0.999
**Sodium acetylsalicylate**	296	0.36×10^3	0.999
**Flurbiprofen sodium salt**	247	1.87×10^3	0.999
**Ketoprofen sodium salt**	254	1.39×10^3	0.998
**Naproxen sodium salt**	261	0.49×10^3	0.999
**Paracetamol**	247	1.24×10^3	0.998

Sodium acetylsalicylate

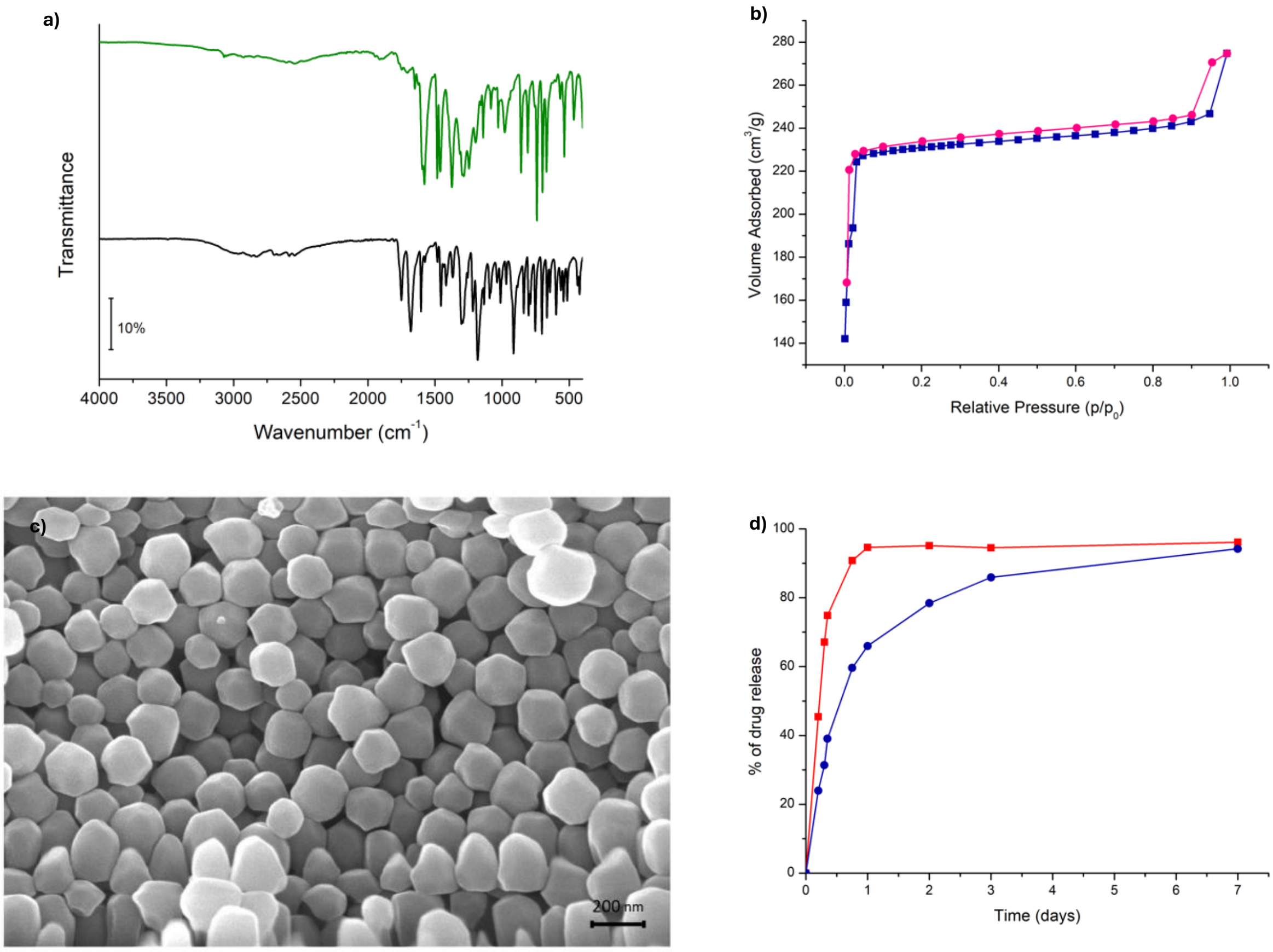

Fig. S2. a) ATR-MIR spectra of acetyl salicylic acid (black) and sodium acetylsalicylate (green). b) N_2 adsorption (blue)-desorption (red) isotherms at 77 K of sodium acetylsalicylate@ZIF-8. c) SEM image collected at 50 kX of the sodium acetylsalicylate@ZIF-8 system. d) time-dependent release rate trend of sodium acetylsalicylate at pH 5.5 (red line) and pH 7.4 (blue line).

Flurbiprofen sodium salt

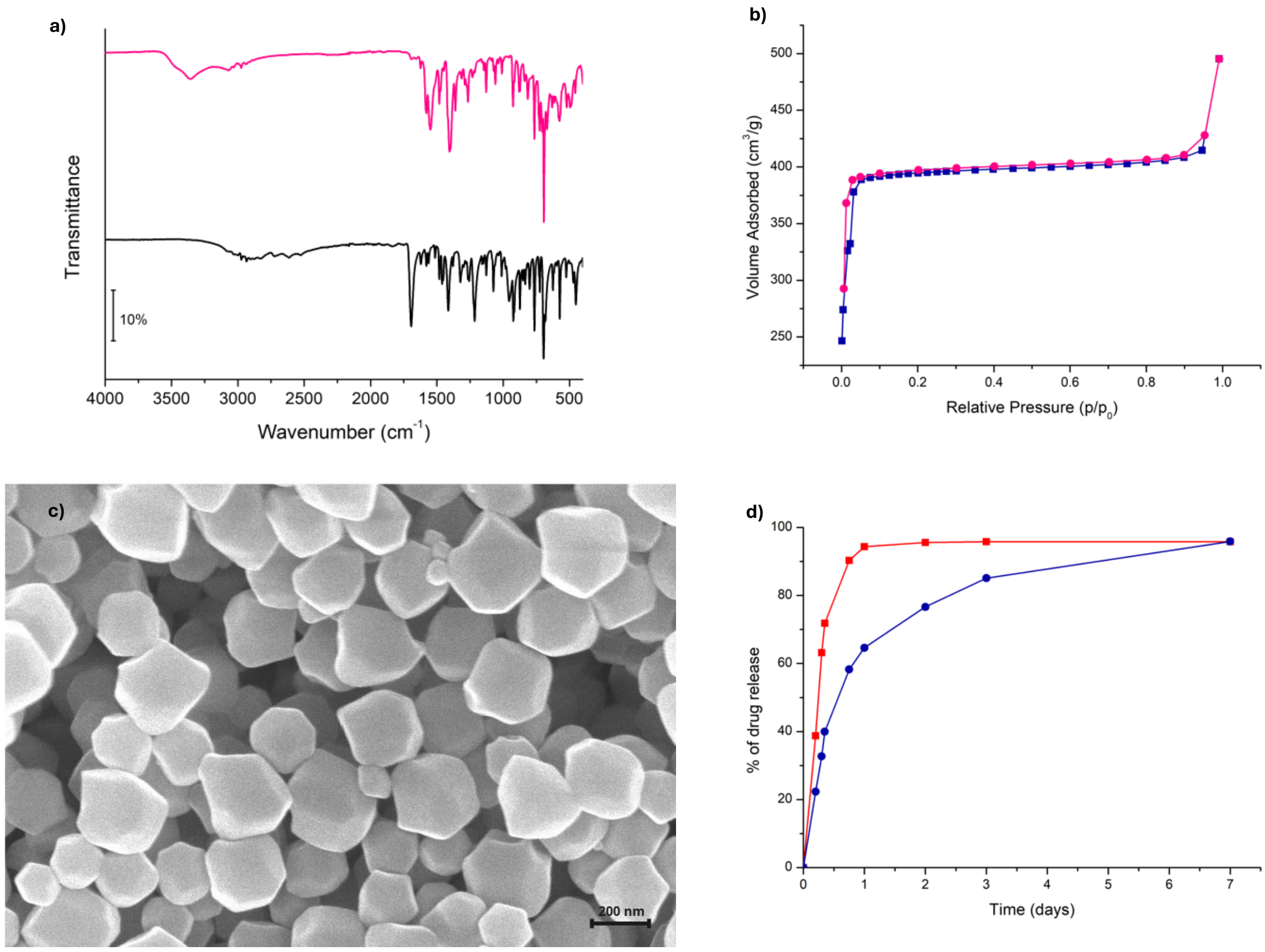

Fig. S3. a) ATR-MIR spectra of flurbiprofen (black) and flurbiprofen sodium salt (magenta). b) N_2 adsorption (blue)-desorption (red) isotherms at 77 K of flurbiprofen sodium salt@ZIF-8. c) SEM image collected at 50 kX of the flurbiprofen sodium salt@ZIF-8 system. d) time-dependent release rate trend of flurbiprofen sodium salt at pH 5.5 (red line) and pH 7.4 (blue line).

Ketoprofen sodium salt

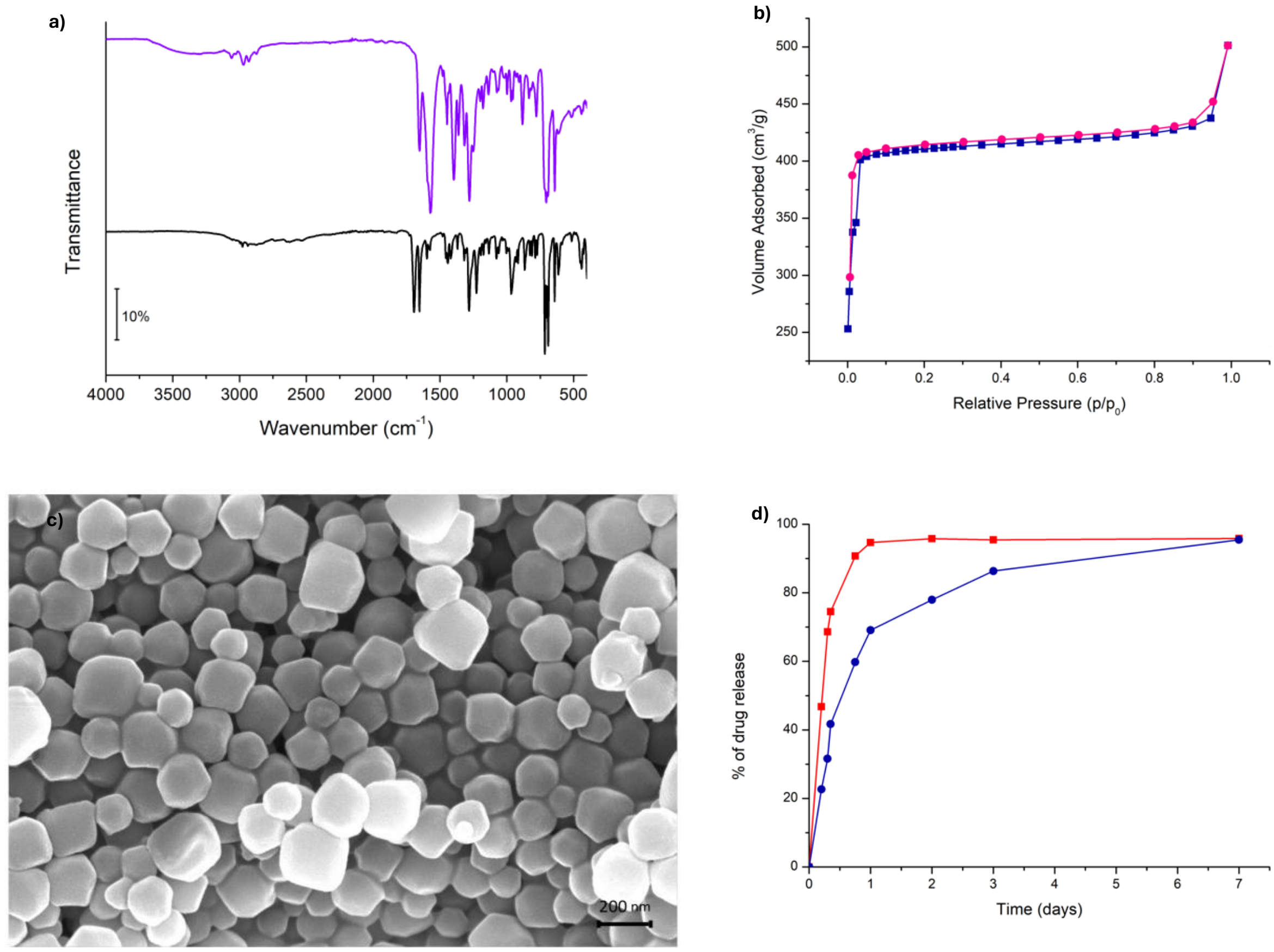

Fig. S4. a) ATR-MIR spectra of ketoprofen (black) and ketoprofen sodium salt (purple). b) N_2 adsorption (blue)-desorption (red) isotherms at 77 K of ketoprofen sodium salt@ZIF-8. c) SEM image collected at 50 kX of the ketoprofen sodium salt@ZIF-8 system. d) time-dependent release rate trend of ketoprofen sodium salt at pH 5.5 (red line) and pH 7.4 (blue line).

Naproxen sodium salt

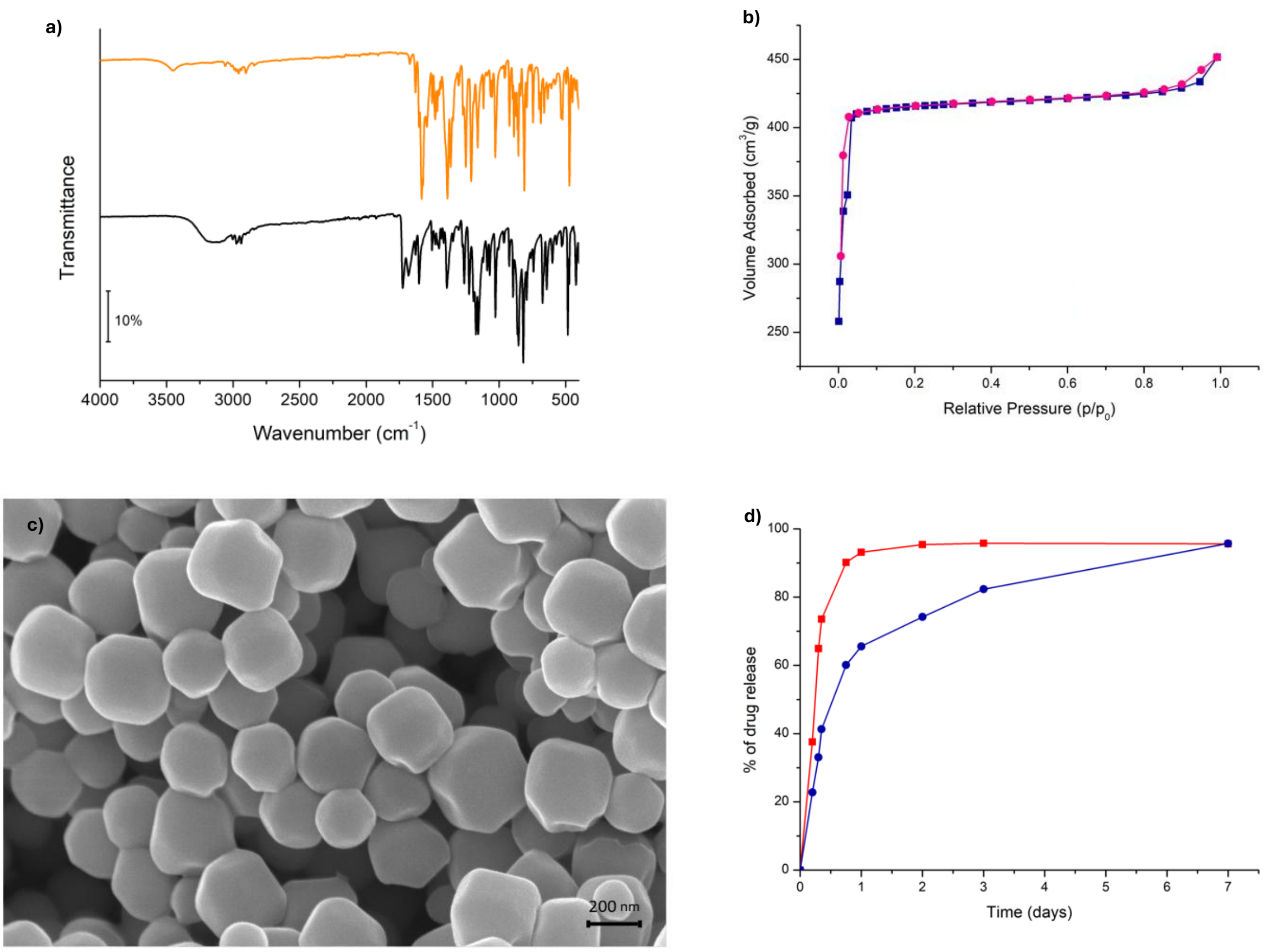

Fig. S5. a) ATR-MIR spectra of naproxen (black) and naproxen sodium salt (orange). b) N_2 adsorption (blue)-desorption (red) isotherms at 77 K of naproxen sodium salt@ZIF-8. c) SEM image collected at 50 kX of the naproxen sodium salt@ZIF-8 system. d) time-dependent release rate trend of naproxen sodium salt at pH 5.5 (red line) and pH 7.4 (blue line).

Paracetamol

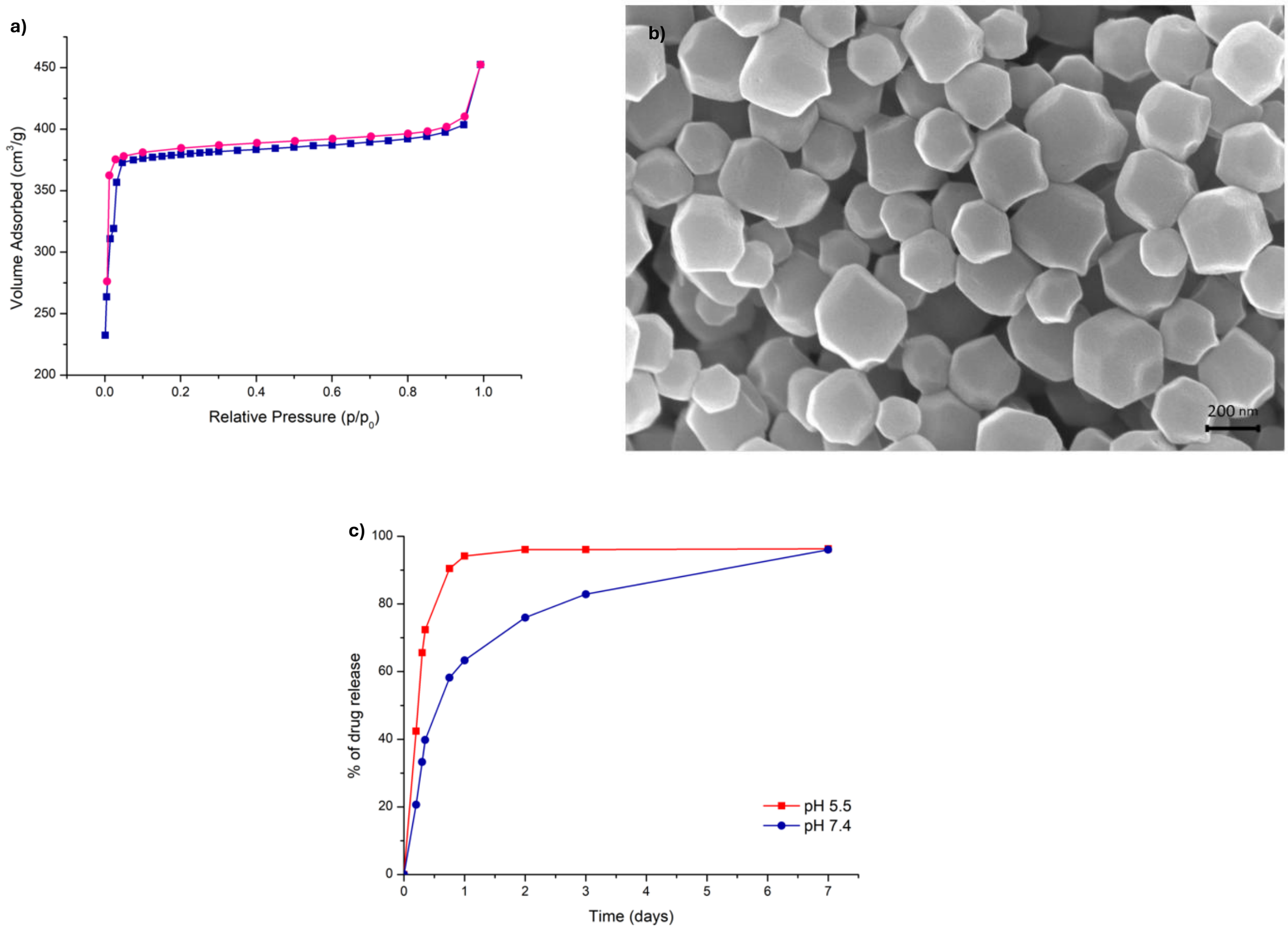

Fig. S6. a) N_2 adsorption (blue)-desorption (red) isotherms at 77 K of paracetamol@ZIF-8. b) SEM image collected at 50 kX of the paracetamol@ZIF-8 system. c) time-dependent release rate trend of paracetamol at pH 5.5 (red line) and pH 7.4 (blue line).

Table S2. BET surface area for the as-prepared ZIF-8 and the drug loaded ones.

	**BET surface area (m^2/g)**
**ZIF-8**	1837
**Ibuprofen sodium salt@ZIF-8**	1447
**Sodium acetylsalicylate@ZIF-8**	838
**Flurbiprofen sodium salt@ZIF-8**	1359
**Ketoprofen sodium salt@ZIF-8**	1511
**Naproxen sodium salt@ZIF-8**	1573
**Paracetamol@ZIF-8**	1390

Biological test

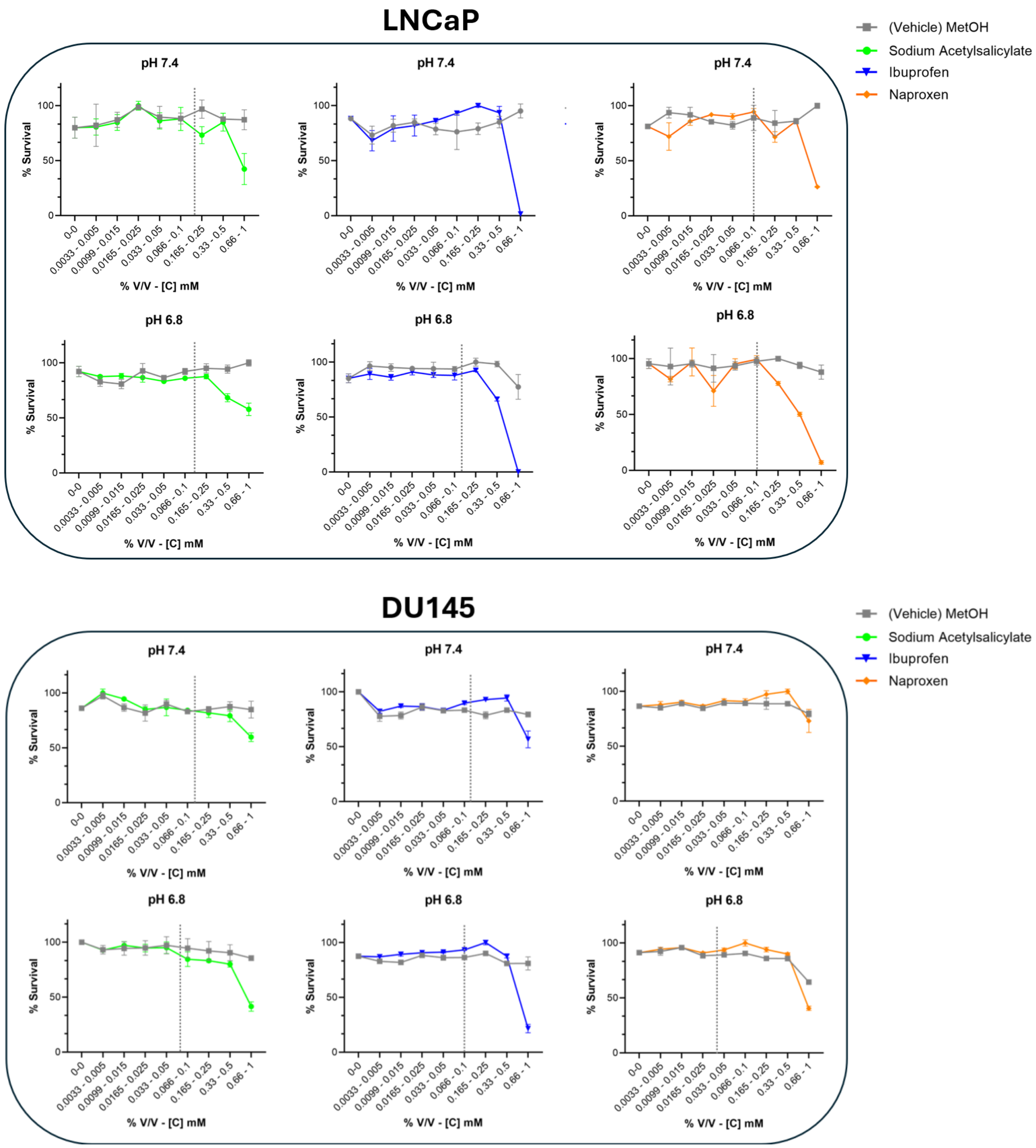

Fig. S7. PrestoBlue Viability Assay on LNCaP (top) and DU145 (bottom) prostate cancer cell lines treated with free drugs. Concentrations of MOF-delivered drugs that reduce cell viability under 50% in Fig. 5 are indicated in this Figure with dotted gray lines.
